# ATF3 Stimulates IL-17A by Regulating Intracellular Ca^2+^/ROS-Dependent IL-1β Activation During *Streptococcus pneumoniae* Infection

**DOI:** 10.3389/fimmu.2018.01954

**Published:** 2018-08-30

**Authors:** Seungyeop Lee, Gyu-Lee Kim, Na Young Kim, Se-Jin Kim, Prachetash Ghosh, Dong-Kwon Rhee

**Affiliations:** ^1^School of Pharmacy, Sungkyunkwan University, Suwon, South Korea; ^2^Department of Life Sciences, Korea University, Seoul, South Korea

**Keywords:** *Streptococcus pneumoniae*, ATF3, IL-17A, IL-1β, NLRP3, inflammasome, γδ T cells, bone marrow-derived macrophages

## Abstract

Activating transcription factor-3 (ATF3) in the ER stress pathway induces cytokine production and promotes survival during gram-positive bacterial infection. IL-17A is a critical cytokine that is essential for clearance of *Streptococcus pneumoniae*. However, the mechanism by which ATF3 induces IL-17A production remains unknown. Here, we show that ATF3 induces IL-17A production via NLRP3 inflammasome-dependent IL-1β secretion. Survival rates were comparable in IL-17A-depleted and ATF3 KO mice but were lower than in WT mice treated with isotype control, indicating that ATF3 positively regulated IL-17A production. Indeed, ATF3 KO mice showed a marked reduction in IL-17A protein and mRNA expression compared to levels in WT mice. Moreover, mitochondrial IL-1β production by bone marrow-derived macrophages was significantly reduced in ATF3 KO mice as a result of the disruption of cellular ROS and Ca^2+^ homeostasis. Accordingly, ATF3 KO mice displayed diminished survival and bacterial clearance following *S. pneumoniae* infection. Taken together, these data suggest a mechanism in which macrophage ATF3 promotes IL-17A production in γδ T cells to rapidly induce host defenses during early *S. pneumoniae* infection.

## Introduction

Activating transcription factor-3 (ATF3) is a fundamental transcription factor in the endoplasmic reticulum (ER)-oxidative stress pathway that acts as a super-enhancer in macrophages (1, 2). During bacterial sepsis, the host responds to infection by upregulating or suppressing cytokines via ATF3 (3, 4). In the case of gram-negative bacteria, ATF3 attenuates pro-inflammatory cytokine production during *Escherichia coli* and *Neisseria gonorrhoeae* invasion (5, 6). As such, ATF3 KO mice exhibit prolonged survival over WT controls following infection with gram-negative bacteria owing to the induction of ATF3-mediated sepsis-associated immunosuppression (3). Thus, the anti-inflammatory effects of ATF3 during gram-negative bacterial infection have been reported (7, 8). In addition, it has been reported that ATF3 enhances pro-inflammatory cytokine production in response to infection by gram-positive pathogens such as *Streptococcus pneumoniae, Listeria monocytogenes*, and *Staphylococcus aureus* (9). However, the functional significance of ATF3 in gram-positive infection remains poorly understood.

IL-17A is secreted by γδ T cells, innate lymphoid cells, and Th17 cells in the mucosal tissue and plays a central role in the innate host defense against various bacteria, such as *Mycobacterium tuberculosis* and *S. aureus* (10, 11). γδ T cells are rapidly stimulated by cytokines secreted by activated macrophages (12). Although γδ T cell-derived IL-17A plays known roles in bacterial clearance, its role in lung bacterial defenses is still not clear.

*S. pneumoniae* is gram-positive bacterium that causes pneumonia, meningitis, and sepsis in humans, resulting in a 10–19% mortality rate. Moreover, the introduction of pneumococcal vaccines has resulted in the advent of antibiotic-resistant strains due to efficient genetic transformation and the rapid accumulation of genetic variation (13). According to clinical research, IL-17A is the most critical cytokine in the early defense against pneumococci. Interestingly, however, IL-17A is nearly undetectable in naïve infants, and vaccination increases IL-17A levels (14). Mice deficient in IL-17A and IL-17RA demonstrate increased susceptibility to a large number of bacterial pathogens that cause lung diseases (15, 16). Even so, the mechanism of IL-17A production during *S. pneumoniae* infection is largely unknown. In the present study, ATF3 KO mice infected with *S. pneumoniae* showed a decrease in γδ T cell-derived IL-17A secretion, as well as macrophage IL-1β levels. Given that ATF3 also plays an important role in NLRP3 inflammasome-dependent IL-1β secretion, these data support the idea that ATF3 is an important inflammatory factor in early pneumococcal infection.

## Materials and methods

### Animals

Six-to-eight-week-old female C57BL/6 WT mice and C57BL/6 ATF3 KO mice were obtained from Orient Bio, Inc. (Seoul, Korea) and Dr. Tsonwin Hai (Ohio State University, Columbus, OH, USA), respectively. Mouse genotyping was performed using PCR as previously described (17). Animals were maintained under controlled temperature and humidity with 12-h light/dark cycles and open access to food and water. This study was carried out in accordance with the guidelines of the Korean Academy of Medical Sciences. The protocol was approved by the Ethics Committee of SungKyunKwan University.

### Bacterial culture

*S. pneumoniae* D39 (serotype 2; Prof. David E. Briles, University of Alabama at Birmingham, Birmingham, AL, USA) (18) was cultured in sterile 3% Todd Hewitt Broth with 0.5% yeast extract in deionized water. *Klebsiella pneumoniae* ATCC 9997 (Korean Culture Center of Microorganisms, Seoul, Korea) was cultured in Difco Nutrient Broth (Difco, Franklin Lakes, NJ, USA). The uropathogenic *E. coli* (UPEC) CFT073 and methicillin-resistant *S. aureus* USA300 were cultured in Luria-Bertani broth (BD, Franklin Lakes, NJ, USA). All bacteria were inoculated and cultured at 37° C (19).

### Mouse model of *S. pneumoniae* infection

Female WT mice were subjected to immune cell depletion or cytokine neutralization with anti-mouse TCR γ/δ (BE0070, Bio X Cell, West Lebanon, NH, USA), anti-mouse/rat IL-1β (BE0246, Bio X Cell), anti-mouse IL-23p19 (16-7232-81, eBiosceince, San Diego, CA, USA), or polyclonal Armenian hamster IgG (BE0091, Bio X Cell) as previously described (20). Alternatively, IL-17A was neutralized in mice using anti-mouse IL-17A (BE0173, Bio X Cell), and the results were compared to those obtained using control anti-mouse IgG1 (BE0083, Bio X Cell) (21). The anesthetized mice were then intranasally inoculated with 2 × 10^8^ colony-forming units (CFU) of bacteria diluted in 30 μL of 0.9% saline (22). The infected mice were monitored every 24 h and then euthanized at 10 days to harvest lung tissue. Bacterial colonization was subsequently assessed using lung homogenates by serial dilution plating on 1.5% THY agar containing 5% sheep blood.

### Immunohistochemistry

Formalin-fixed lung tissue samples were stained with anti-ATF3 (ab180842, Abcam, Cambridge, UK) and anti-iNOS (ab49999, Abcam) and then imaged with an Olympus BX50 light microscope (Olympus, Tokyo, Japan).

### Bone marrow-derived macrophage (BMDM) isolation and infection

WT and ATF3-KO BMDMs were prepared as previously described using L929-conditioned medium (23) and maintained in a 37°C humidified incubator with 5% CO_2_. Cells were treated with 2 mM ATP for 40 min prior to infection with *S. pneumoniae, K. pneumoniae, E. coli*, or *S. aureus* at MOI 20 for 4 h. Alternatively, cells were pretreated with the mitochondrial calcium uptake inhibitor Ru360 (10 μM, #557440, Merck KGaA, Darmstadt, Germany) or vehicle control for 30 min prior to infection with *S. pneumoniae* for 4 h.

### Immunoblotting

Mouse lung samples and BMDMs were homogenized with lysis buffer (Abcam) supplemented with protease and phosphatase inhibitors (78442, ThermoFisher), and the proteins were quantified. Samples were separated by SDS-PAGE and transferred to PVDF membranes using a Trans-Blot Turbo Transfer System (Bio-Rad). The membranes were blocked with skim milk for 2 h, and then probed overnight with anti-GBP5 (gtx31537, Gentex, Zeeland, MI, USA), anti-iNOS (NB300-605, Novus International, St. Louis, MO, USA), anti-IL-1β (AF-401-SP, R&D Systems, Minneapolis, MN, USA), or anti-β-actin (sc-47778, Santa Cruz Biotechnology, Dallas, TX, USA). After washing with TBST, the blots were incubated with the appropriate anti-mouse, anti-goat, or anti-rabbit secondary antibody and then detected with ECL solution (GenDEPOT, Barker, TX, USA). Semi-quantitative densitometry was performed using ImageJ 2.1.4.6 software (National Institutes of Health, Bethesda, MD, USA).

### RNA expression analysis and sequencing

RNA was isolated from mouse lung samples using the RNeasy Plus Mini Kit (74134, Qiagen, Hilden, Germany) and was reverse-transcribed using cDNA EcoDry Premix (639546, Takara, Tokyo, Japan). Expression analysis was performed using a StepOne Real-Time PCR system (Applied Biosystems, Foster City, CA, USA) with the following primers: *Nlrp3* forward, 5′-AATGCCCTTGGAGACACAGGA-3′, *Nlrp3* reverse, 5′-TGAGGTGAGGCTGCAGTTGTCTA-3′; *Gbp5* forward, 5′-TTGAGGCAAATAGCATTGGAGA-3′; *Gbp5* reverse, 5′-CATGTGTTGGAGCTGCTGTTGA-3′; *Il17a* forward, 5′-GAAGGCCCTCAGACTACCTCAA-3′; *Il17a* reverse, 5′-TCATGTGGTGGTCCAGCTTTC-3′; *Il18* forward, 5′-ACCTCCAGCATCAGGACAAAG-3′; *Il18* reverse 5′-TGTACAGTGAAGTCGGCCAAAG-3′.

RNA libraries of lung samples collected from WT and ATF3-KO mice were prepared using SENSE 3′ mRNA-Seq Library Prep Kit (Lexogen, Inc., Austria) according to the manufacturer's instructions and sequenced with a NextSeq 500 system (Illumina, Inc., San Diego, CA, USA). Data were analyzed in R using Bowtie2 version 2.1.0 and DAVID (24). Gene clustering (Hierarchical clustering) and heat maps were constructed based on MeV 4.9.0. This experimental and system biology analyses were analyzed using Ingenuity Pathway Analysis. The gene expression analyses data was deposited in the NCBI database [GEO accession number GSE118195] (http://www.ncbi.nlm.nih.gov/geo/).

### Cytokine elisa

Mouse IL-1β (88-7013-76, Invitrogen, Carlsbad, CA, USA), IL-23 (88-7230-22, eBioscience), s100a8 (MBS2504318, Mybiosourse), s1009a (MBS2886839, Mybiosourse, San Diego, CA, USA), procalcitonin (PCT) (CSB-E10371m, Cusabio, College Park, MD, USA), and G-CSF (MCS00, R&D Systems) ELISA kits were used to assess cytokine levels in lung homogenates. All analyses were performed according to the manufacturer's instructions.

### ATP, Ca^2+^, and ROS measurement

BMDMs (0.5 × 10^6^ cells/mL) seeded in 96-well plates were analyzed for ATP, Ca^2+^, and ROS levels using the ADP/ATP ratio (ab65313, Abcam), Fluo-4 NW calcium (F36206, Invitrogen) and ROS/RNS (STA-347, Cell Biolabs, San Diego, CA, USA) assay kits according to the manufacturer's instructions.

### Flow cytometry

Lungs samples from WT and ATF3-KO mice at 6 h post-infection (hpi) and non-infected controls (*n* = 5 per group) were digested in collagenase as previously described to collect live cells (25). Isolated cells were stained with anti-mouse/rat IL-17A-APC (12-7101, eBioscience) and anti-mouse TCR gamma/delta-PE (12-5711-82, eBioscience) (20) and analyzed with a Guava EasyCyte flow cytometer (EMD Millipore, Billerica, MA, USA) and FCS Express 6 software (De Novo Software, Boulder, CO, USA).

### Statistical analysis

All data were collected from at least three independent experiments. Differences between groups were analyzed by Mann–Whitney rank sum test and one- or two-way ANOVA. Survival analysis was performed using log-rank testing. Statistical significance was defined as *P* < 0.05.

## Results

### ATF3 activates NLRP3 inflammasome, IL-1β secretion, and subsequent IL-17A production

**Figure 1:**
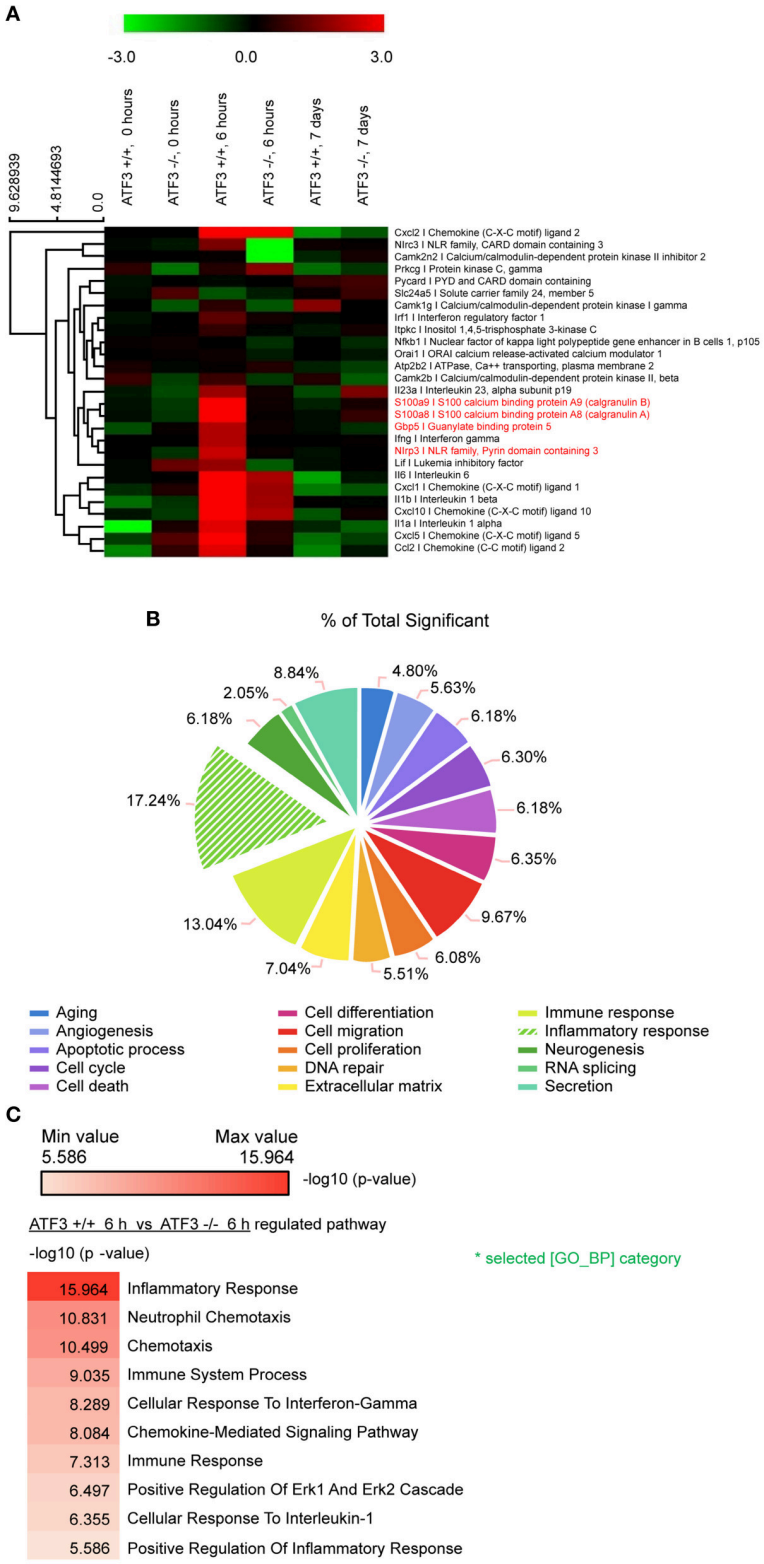
ATF3 promotes inflammatory gene expression during early *S. pneumoniae* infection. **(A)** Expression heat map. Red and green represent up- and down-regulation, respectively. Genes associated with macrophage inflammation are shown. **(B)** Classification of genes with significant expression differences between WT and ATF3 KO mice. **(C)** Pathways enriched in WT mice at 6 hpi as compared to ATF3 KO counterparts.

To understand how ATF3 mediates the regulation of related genes after pneumococcal infection, RNA-seq analysis was performed of lung mRNAs from WT and ATF3 KO mice. The RNA-seq results showed that ATF3 regulated various pathways during the early (6 hpi) and late (7 days) stages (Figure 1A). In particular, ATF3 played a major role in the inflammatory response (Figure 1B) by mediating neutrophil chemotaxis, ERK signaling, and the cellular responses to IFN-γ and IL-1 (Figure 1C). An increase in the cellular response to IFN-γ reportedly facilitates NLRP3 inflammasome assembly via IRF/guanylate-binding protein 5 (GBP5) signaling (26), suggesting that ATF3 affects NLRP3 inflammasome activation and controls IL-1β secretion and subsequent IL-17A production (12) to participate in the host defense against *S. pneumoniae*.

### ATF3 promotes NLRP3 inflammasome activation

**Figure 2:**
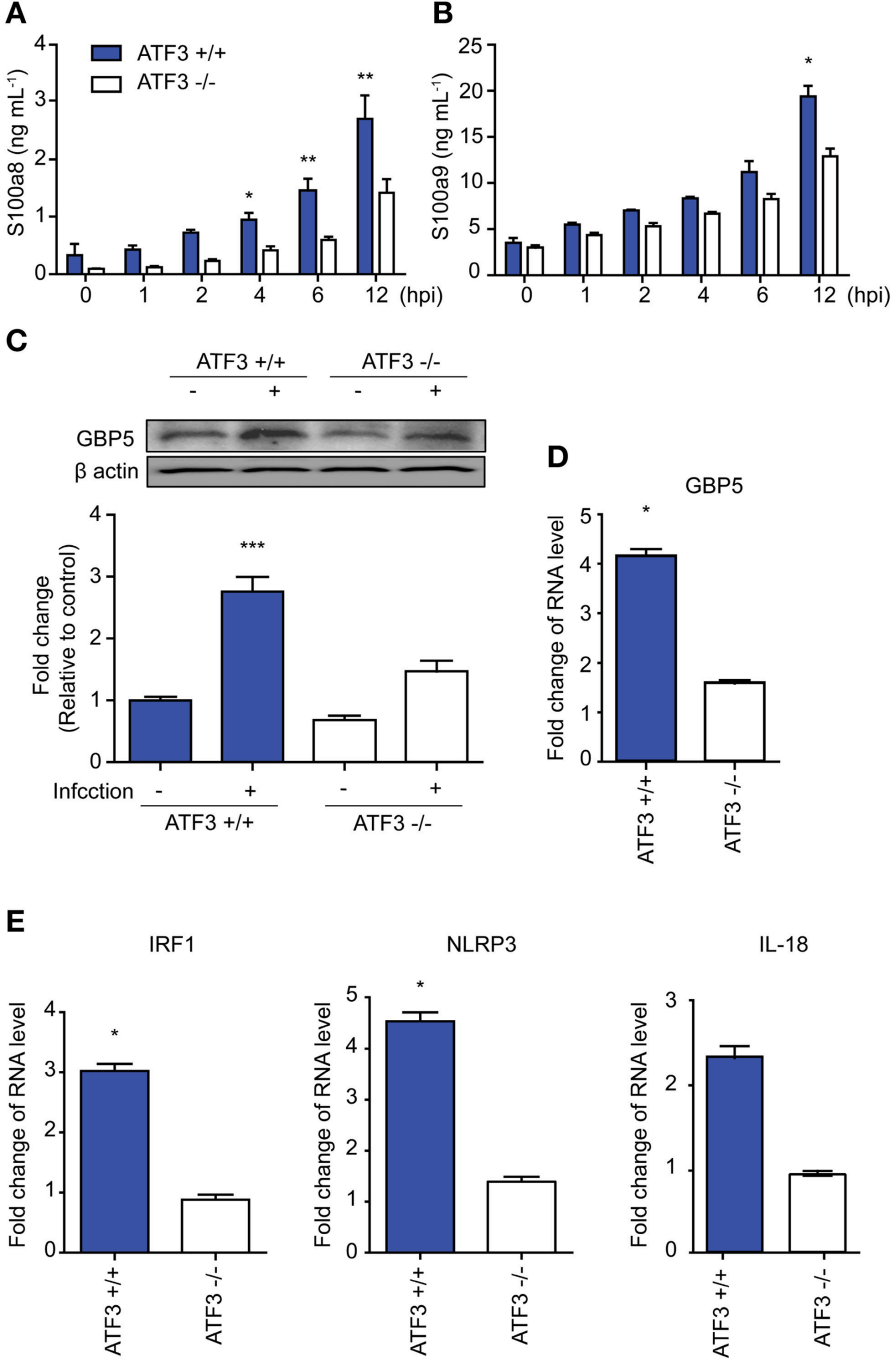
ATF3 is involved in NLRP3 inflammasome activation. **(A)** s100a8 and **(B)** s100a9 expression was examined at 0, 1, 2, 4, 6, and 12 hpi in bronchoalveolar lavage fluid (BALF) samples. **(C)** GBP5 expression at the protein and **(D)** mRNA levels at 0 and 6 hpi. **(E)** *Irf1, Nlrp3*, and *Il18* mRNA expression at 6 hpi. Data are presented as the mean ± SD of triplicate samples from at least three independent experiments. ^*^p < 0.05, ^**^p < 0.01, ^***^p < 0.001, two-way analysis and *t*-test of variance.

To confirm our RNA-seq findings, IL-1β target gene expression was examined in WT and ATF3 KO mice infected with *S. pneumoniae*. The cytokines S100a8 and S100a9 are produced by phagocytes and induce IL-1β secretion (27). Both S100a8 and S100a9 levels were significantly lower in the bronchoalveolar lavage fluid (BALF) samples of ATF3 KO mice at 4 hpi than in WT BALF samples (Figures 2A,B). GBP5 is the factor that assembles the NLPR3 inflammasome in response to bacteria (28). As expected, GBP5 expression was significantly induced in WT mice at 6 hpi (Figures 2C,D). Furthermore, *Irf1, Nlrp3*, and *Il18* mRNA expression was higher in the lungs of WT mice at 6 hpi than in those of ATF3 KO mice, consistent with our RNA-seq data (Figure 2E). These results further support the idea that ATF3 plays a major role in NLRP3 inflammasome signaling.

**Figure 3:**
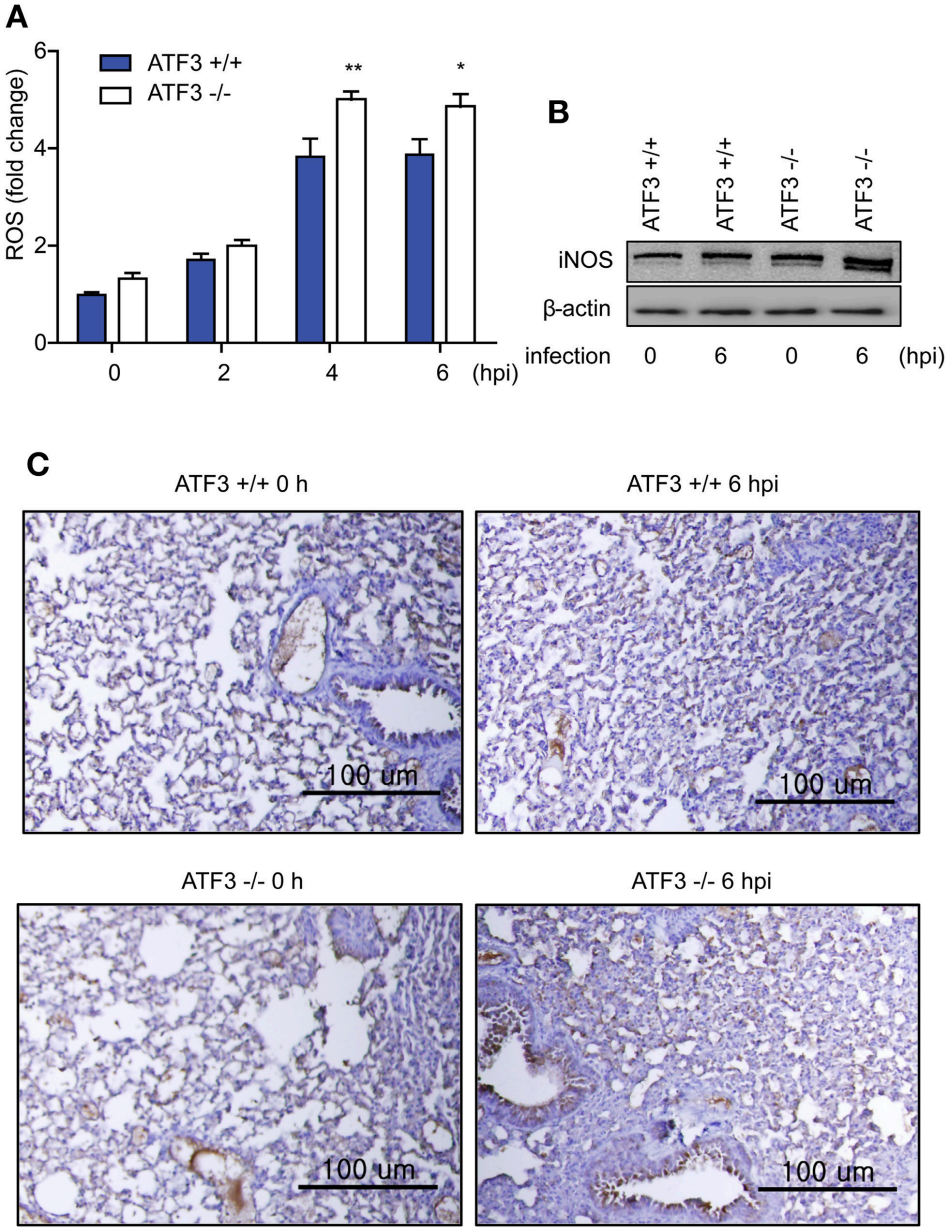
ATF3 suppresses ROS generation and iNOS expression. **(A)** Fold change in ROS levels at 0, 2, 4, and 6 hpi compared to that in non-infected WT cells. **(B)** iNOS expression and **(C)** IHC (anti-iNOS) at 0 and 6 hpi (*n* = 4). ^*^P < 0.05, ^**^P < 0.01, two-way analysis of variance.

Recently, it has been reported that ROS-derived iNOS inhibits NLRP3 inflammasome activity (29). We next measured the levels of ROS and iNOS in lungs after *S. pneumoniae* infection to validate the functional significance of ATF3 in NLRP3 inflammasome activation. Notably, ROS levels continuously increased in WT infected lungs until 4 hpi, and ROS levels were significantly higher in ATF3 KO lungs than in WT lungs after 4 hpi (Figure 3A), confirming that ATF3 KO mice exhibited increased iNOS expression (Figure 3B). Moreover, at 6 hpi, IHC staining for iNOS was stronger in *S. pneumoniae*-infected ATF3 KO mouse lungs than in WT mouse lungs (Figure 3C). Collectively, these results indicate that iNOS overexpression inhibits inflammasome activation in ATF3 KO mice. Thus, ATF3 represses iNOS expression by suppressing ROS overproduction in the development of NLRP3 inflammasome.

### ATF3 is critical in the host response to early *S. pneumoniae* infection

**Figure 4:**
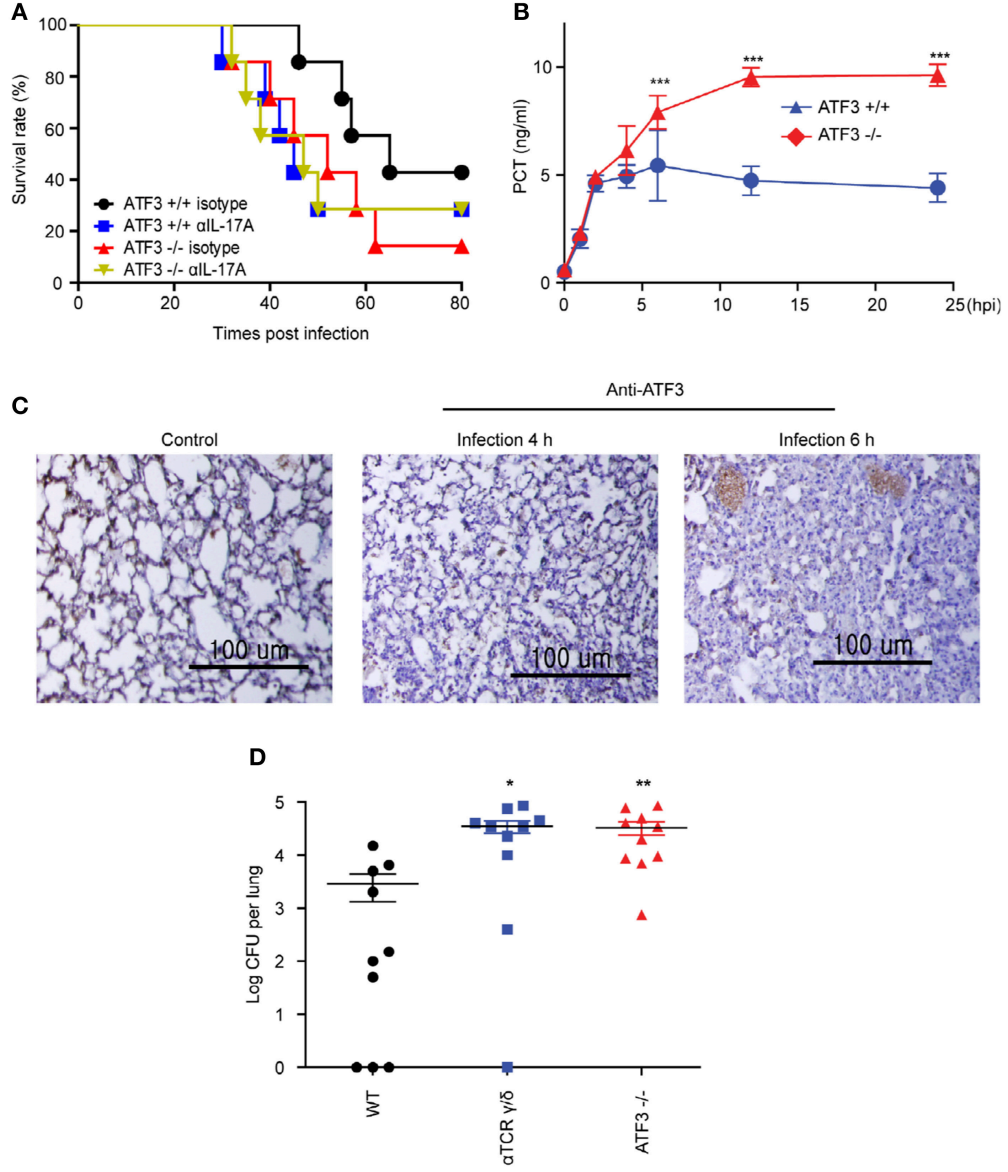
ATF3 and IL-17A promote early immune defenses against *S. pneumoniae* infection. **(A)** Survival rates of WT and ATF3 KO mice treated with IL-17A or isotype control antibodies after *S. pneumoniae* infection (*n* = 7). **(B)** Procalcitonin (PCT) levels in WT and ATF3 KO mice at 0, 1, 2, 4, 6, 12, and 24 h post-infection (hpi) (*n* = 4). **(C)** ATF3 immunostaining in WT lungs after 0, 4, or 6 hpi (*n* = 3). **(D)** *S. pneumoniae* colonization was analyzed in lung homogenates obtained from WT, γδT cell-depleted WT, and ATF3-KO mice (*n* = 10). Data are presented as the mean ± SD from at least three independent experiments. Mouse survival was analyzed by log-rank test. ^*^p < 0.05, ^**^p < 0.01, ^***^p < 0.001, two-way analysis and *t*-test of variance.

ATF3 KO mice showed a marked increase in mortality following *S. pneumoniae* infection as compared to that in WT controls (4). γδ T cells are the primary mediators of IL-17A secretion in the innate immune system (12). We tested whether the correlation of ATF3 with IL-17A secretion by γδ T cells was a key factor in the early host defense to *S. pneumoniae* infection. IL-17A was depleted using anti-IL-17A neutralizing antibody (αIL-17A). Notably, αIL-17A-treated mice displayed a much higher mortality rate post-infection than WT mice. However, there was no significant effect of IL-17A neutralizing antibody in ATF3 KO mice (Figure 4A). To further determine whether ATF3 contributes to sepsis, levels of the sepsis marker procalcitonin were determined. Levels of procalcitonin were significantly higher in WT mice than in ATF3 KO mice at 6 hpi (Figure 4B). In addition, ATF3 expression was rapidly induced in the lung at 4 and 6 hpi (Figure 4C), suggesting that ATF3 plays an important role in sepsis via IL-17A.

### Lung γδ T cells produce IL-17A in response to ATF3-dependent IL-1β secreted by macrophages

**Figure 5:**
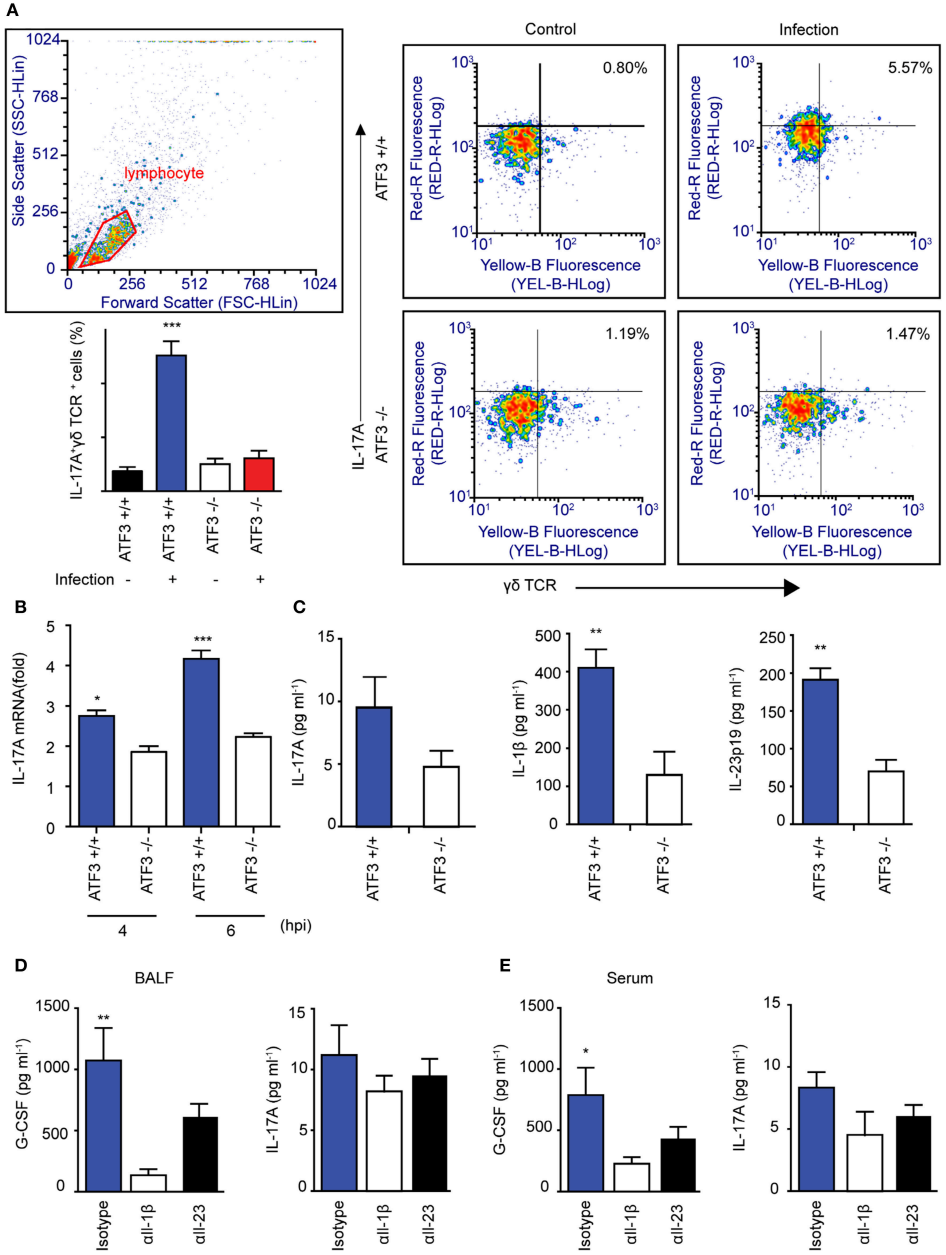
Association between macrophage ATF3 and γδ T cell IL-17A expression. **(A)** IL-17A^+^ γδ+ T cell numbers, **(B)** *Il17a* mRNA expression, and **(C)** IL-17A, IL-1β, and IL-23p19 levels in the lungs of infected mice (*n* = 4 each) as a fold change relative to that in uninfected mice. **(D)** Cytokine levels in bronchoalveolar lavage fluid (BALF) samples collected from mice treated with αIL-1β, αIL-23, or isotype control antibody. **(E)** IL-17A and G-CSF levels in serum (*n* = 5). Data are presented as the mean ± SD of triplicate samples from at least three independent experiments. ^*^p < 0.05, ^**^p < 0.01, ^***^p < 0.001, two-way analysis of variance.

To further confirm that ATF3 and γδ T cells are involved in IL-17A production and subsequent bacterial clearance, the bacterial load was determined in ATF3 KO and γδ T cell-depleted mice. ATF3 KO and γδ T cell-depleted WT mice showed a 10-fold increase in bacterial burden compared to that in WT controls (Figure 4D). To confirm IL-17 secretion from γδ T cells, the number of IL-17A^+^ γδ T cells was determined by FACS. As expected, WT lungs showed approximately a 5-fold increase in IL-17A^+^ γδ T cell numbers over their ATF3 KO counterparts (Figure 5A). Moreover, lung *Il17a* mRNA levels were significantly higher at 4 and 6 hpi in infected WT mice than in infected ATF3 KO mice (Figure 5B). Furthermore, IL-17A, IL-1β, and IL-23p19 levels in BALF samples from WT mice were higher than in those collected from ATF3 KO mice at 6 hpi (Figure 5C). To further check whether this response was mediated by ATF3, mice were treated with anti-IL-1β or anti-IL-23 antibody (αIL1β and αIL23, respectively), and the IL-17A level was assessed. G-CSF is important for IL-17A-mediated granulopoiesis into human lungs (30). Anti-IL-1β- or anti-IL-23-treated WT mice also exhibited lower IL-17A and G-CSF levels than untreated controls after *S. pneumoniae* infection (Figures 5D,E). Taken together, these data indicate that ATF3 likely regulates IL-17 expression in an IL-1β- and IL-23-dependent manner. In contrast, the expression of IL-1β was significantly attenuated in *S. pneumoniae*-infected ATF3 KO mice, suggesting an impairment of IL-17A secretion, which is essential for survival during early *S. pneumoniae* infection.

### ATF3 regulates ROS-Ca^2+^ for IL-1β activation during *S. pneumoniae* infection

**Figure 6:**
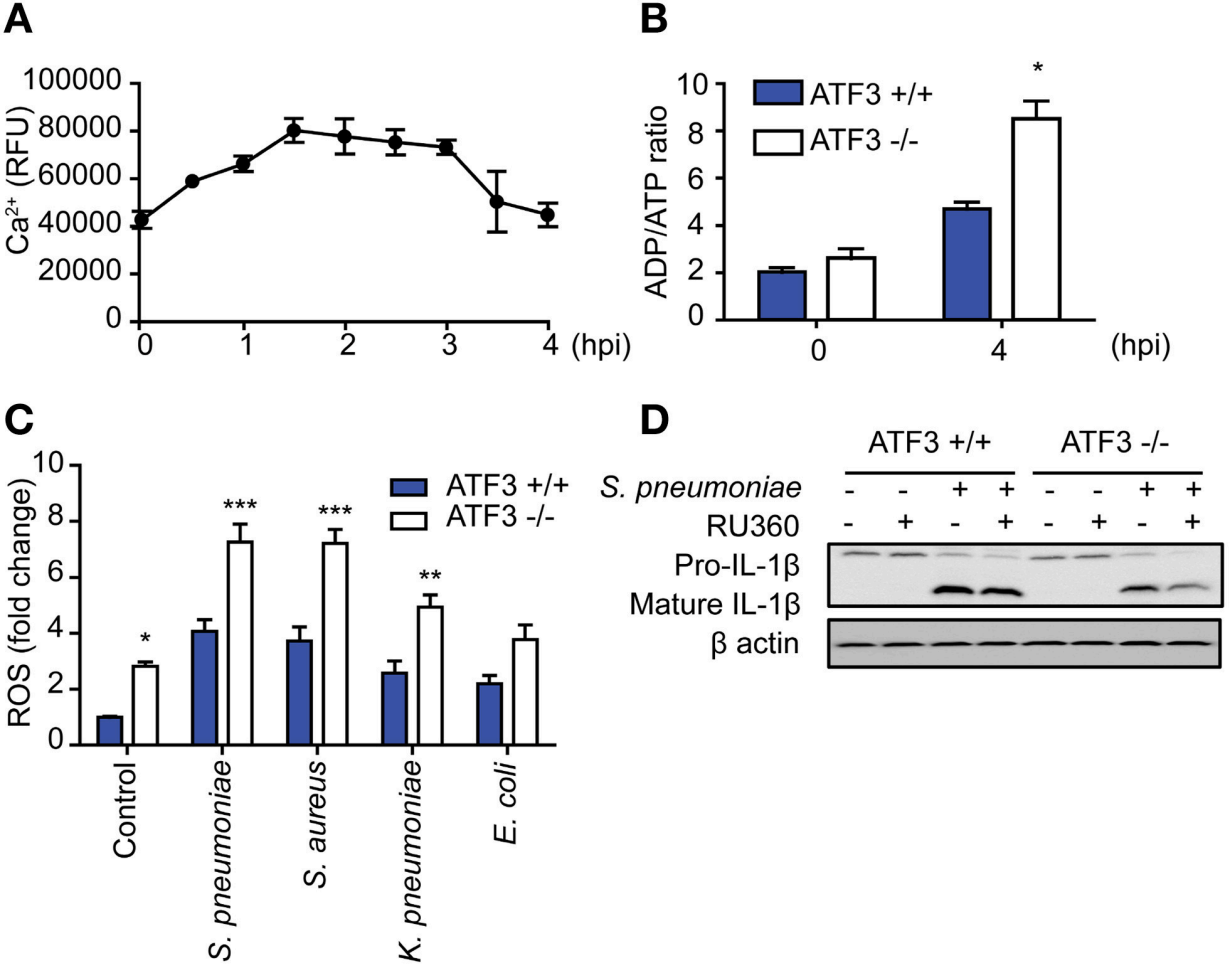
ATF3 induces IL-1β secretion by controlling ROS and Ca^2+^ levels in macrophages during *S. pneumoniae* infection. **(A)** Intracellular calcium levels were monitored at 30-min intervals from 0 to 4 hpi in bone marrow-derived macrophages (BMDMs) infected with *S. pneumoniae* (MOI 20). **(B)** ADP/ATP changes in WT and ATF3 KO cells at 4 hpi. **(C)** Effect of *S. pneumoniae, S. aureus, K. pneumoniae*, and *E. coli* infection (MOI 20) on ROS production at 4 hpi. **(D)** Effect of the calcium uptake inhibitor Ru360 on IL-1β protein expression in WT and ATF3 KO cells. ^*^P < 0.05, ^**^P < 0.01, ^***^P < 0.001, two-way analysis of variance.

ATF3 is an important factor in ER stress downstream of various stimuli (1), during which the Ca^2+^ stored in the ER is transported to the mitochondria together with ROS. Infection-induced ROS induces ER stress and increases intracellular Ca^2+^ levels (31). To further determine the role played by ATF3 in *S. pneumoniae*-induced activation of IL-1β, intracellular Ca^2+^ levels were measured in *S. pneumoniae*-infected bone marrow-derived macrophages (BMDMs). Intracellular Ca^2+^ levels gradually increased after pneumococcal infection in a time-dependent manner and later decreased after 3 hpi (Figure 6A). Intracellular Ca^2+^ typically mediates mitochondria-dependent ATP production, which is essential for IL-1β production (32). ADP/ATP increased more rapidly in ATF3 KO BMDMs than in their WT counterparts (Figure 6B). These data indicate that ATF3 is required for *S. pneumoniae*-induced ATP utilization.

To counteract these events, macrophages utilize ATP-dependent Ca^2+^ pumps on the cell surface to relieve intracellular Ca^2+^ stress and maintain the intracellular calcium balance (33). Procalcitonin, a calcitonin precursor, healthy individuals secrete calcitonin to maintain calcium homeostasis (34). However, the ER stress generated during bacterial infection disrupts Ca^2+^ homeostasis and causes excessive ROS production. This phenomenon either blocks NLRP3 priming or promotes cell death (35, 36). In a previous study, we identified different reactions in cytokinesis dependent on whether infection was caused by gram-positive or -negative bacteria (9). Consistent with cytokines, ATF3 KO BMDMs exhibited increased ROS production after infection with either gram-positive or gram-negative bacteria. However, ROS levels in gram-positive infections, including *S. pneumoniae* and *S. aureus* infection, were higher than those in gram-negative infections (Figure 6C). To determine the effect of ROS-induced Ca^2+^ stress on IL-1β production, BMDMs were treated with the mitochondrial calcium uptake inhibitor RU360 and analyzed for IL-1β processing. ATF3 KO BMDMs with excessive ROS showed a more significant reduction in mature IL-1β levels (Figure 6D). Taken together, these results indicate that ATF3 plays a key role in *S. pneumoniae* infection by promoting IL-1β secretion via ROS and Ca^2+^ crosstalk in macrophages.

## Discussion

**Figure 7:**
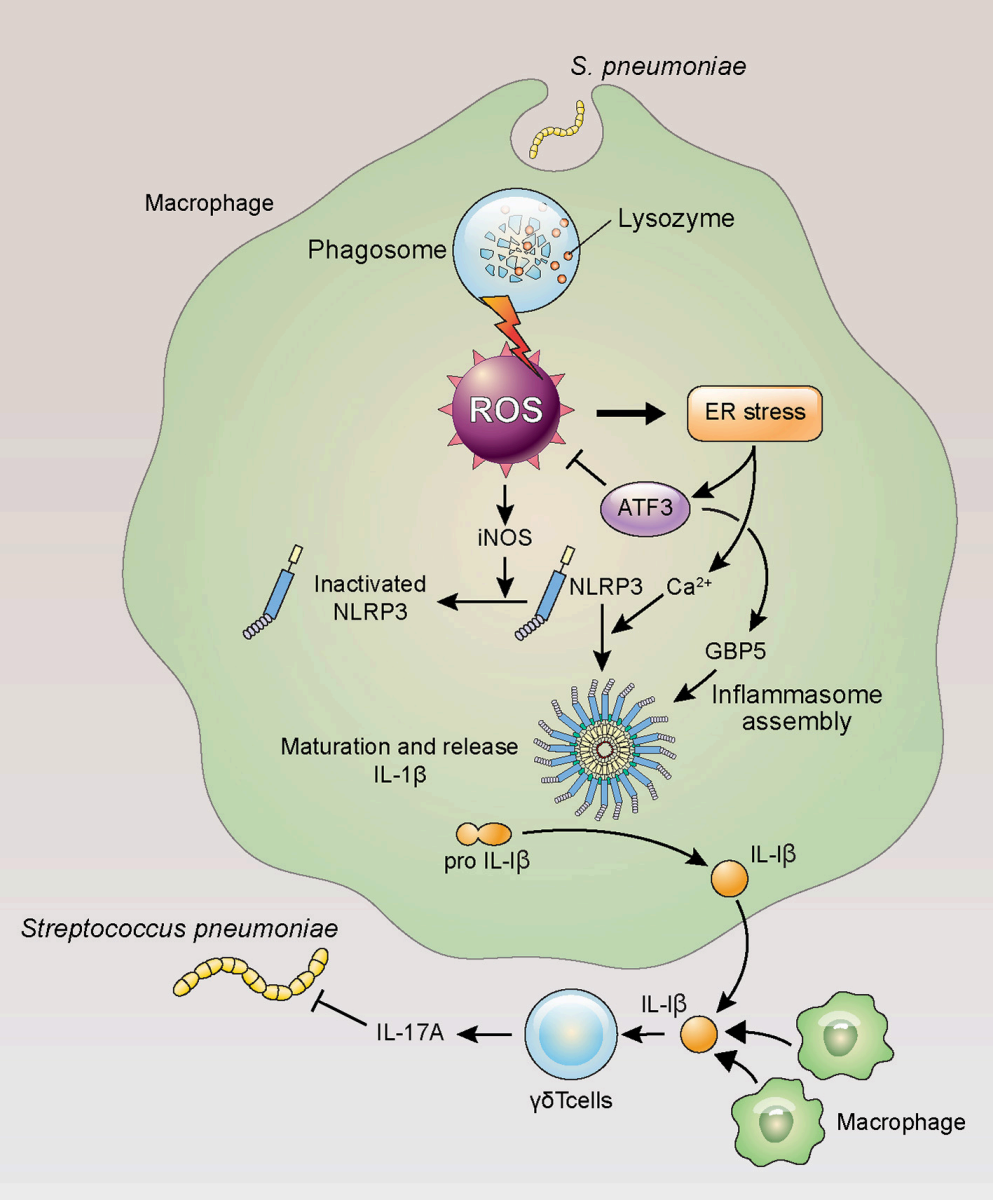
ATF3 plays an important role in IL-17A production in response to *S. pneumoniae* infection. Lung macrophages rapidly phagocytose invading *S. pneumoniae* during infection, resulting in ROS production, ER stress, and ATF3 activation. ATF3 then inhibits ROS-induced iNOS expression to promote NLRP3 inflammasome and GBP5 activation, triggering IL-1β secretion and the subsequent activation of γδ T cells in the lung (39).

In this study, ATF3 deficiency hindered ROS homeostasis in mice, resulting in diminished post-infection survival. Given that ATF3 KO mice displayed elevated procalcitonin levels, these data support the use of this factor as a sepsis biomarker. Interestingly, ROS levels in *E. coli*-infected ATF3 KO mice were similar to those in WT mice infected with gram-positive bacteria with high IL-1β production (Figure 6). In this model, ROS removal had no impact on the survival of ATF3 KO mice, whereas a significant difference was observed in WT mice when ROS were removed (Figure 3). Moreover, WT mouse survival was previously observed to be longer than that of ATF3 KO mice following LPS challenge; however, ATF3 KO mouse survival was significantly higher in mice infected with *E. coli* (3). Accordingly, ATF3 is believed to moderate ROS levels rather than to serve as an unconditional negative regulator in gram-negative bacteria. Thus, it was presumed that the similar ROS levels observed during infection with gram-positive bacteria in ATF3 KO mice resulted from defects in ROS inhibition and cytokine production, and thus failed to defend against *E. coli* through an inability to produce sufficient cytokines (9). In the case of gram-negative bacteria, inflammasome activation elicits IL-1β production via a complicated mechanism (37, 38). Moreover, ROS regulation by ATF3 varies based on bacterial species and infection stage. Therefore, it seems that IL-1β production through ATF3-mediated ROS inhibition is necessary to prevent the inhibition of primed NLRP3, as a sufficient amount of ROS was secreted during infection in the case of gram-positive bacteria (Figure 7).

Macrophages reside in the lungs in large quantities and orchestrate interactions with other immune cells during acute infection (40). Our group previously demonstrated that ATF3 induction in macrophages during *S. pneumoniae* infection upregulates the pro-inflammatory cytokines IFN-γ, TNF-α, and IL-1β (4). Previously, the lung immune system showed almost no change in the αβ T cell (CD4^+^/CD8^+^) population after 14 days of *S. pneumoniae* infection, whereas the amount of γδ T cells increased ~30-fold (41). Although previous studies emphasized the importance of IL-17A and γδ T cells (41), the functional significance of these cells in *S. pneumoniae* infection remains unclear. In the lungs of infected mice, macrophage ATF3 activates the NLRP3 inflammasome to induce IL-1β secretion, which subsequently stimulates IL-17A secretion by γδ T cells.

During ROS-induced ER stress, ATF3 can induce CHOP upregulation to promote IL-23p19 secretion. Alternatively, CHOP downregulation results in increased BCL-2 expression (42), which promotes cell survival and the closing of voltage-dependent anion channels (VDACs) that transport calcium from the outer mitochondrial membrane. When VDAC activity is absent, the NLRP3 inflammasome is not activated and IL-1β secretion is reduced (43). Similarly, mitochondrial-associated endoplasmic reticulum membrane (MEM) protein crosstalk regulates NLRP3 inflammasome activity via ROS (44). Macrophage NLRP3 inflammasome accumulation in response to infection results in IL-1β and IL-18 pro-inflammatory cytokine secretion via ROS and Ca^2+^ (45–47). However, the excessive ER stress generated by infection may cause intracellular Ca^2+^ and ROS disruption that inhibits NLRP3 inflammasome assembly through the ROS–iNOS–NO axis (39, 48, 49). Nevertheless, the mechanism of ER stress-induced NLRP3 activation via Ca^2+^ requires further characterization (50). IL-17A not only is essential for the defense against *S. pneumoniae* but also mediates the early defenses following infection by other gram-positive and gram-negative bacteria (11, 21, 51). In contrast, IL-1β production occurs in a different manner, as the pH of macrophage phagosomes containing gram-positive bacteria is higher than that of gram-negative bacteria. In the case of gram-positive bacteria, higher pH leads to ROS production and NLRP3 inflammasome activation, whereas gram-negative bacteria elicit ROS-independent NLRP3 inflammasome activity (37).

In summary, the present work proposed a pathway in which ATF3 promotes NLRP3 inflammasome activation and assembly by regulating ROS during early *S. pneumoniae* infection. ATF3 also controlled intracellular Ca^2+^ and ATP homeostasis and induced the production of IL-1β and IL-23p19 by macrophages, which stimulated IL-17A secretion by γδ T cells. Thus, ATF3 plays a key role in host survival and pathogen clearance via inflammasome signaling during *S. pneumoniae* infection and may be a potential therapeutic target.

## Author contrubutions

SL and D-KR designed research. SL, G-LK, NK, S-JK, and PG performed research. SL, NK, and D-KR analyzed data and wrote paper. All the authors read and approved the final manuscript.

### Conflict of interest statement

The authors declare that the research was conducted in the absence of any commercial or financial relationships that could be construed as a potential conflict of interest.
